# Effect of calcium lactate supplementation on cholesterol concentration in patients with hyperlipidaemia and previous viral hepatitis: a preliminary report

**Published:** 2008-04

**Authors:** G Andryskowski, J Chojnowska-Jezierska, M Broncel, M Barylski, M Banach

**Affiliations:** Department of Internal Diseases and Unit of Clinical Pharmacology and Monitored Therapy, Medical University of Lodz, Poland; Department of Internal Diseases and Unit of Clinical Pharmacology and Monitored Therapy, Medical University of Lodz, Poland; Department of Internal Diseases and Unit of Clinical Pharmacology and Monitored Therapy, Medical University of Lodz, Poland; Department of Internal Diseases and Cardiological Rehabilitation, Medical University of Lodz, Poland; Department of Cardiology, Medical University of Lodz, Poland

## Abstract

**Summary:**

The aim of the study was to estimate the effect of calcium supplementation on cholesterol concentrations in patients with hyperlipidaemia and previous viral hepatitis.

The study comprised 43 patients, aged 28 to 82 years (21 with type 2 hyperlipidaemia). The control group included 22 healthy subjects. After four weeks of a hypolipaemic diet (wash-out period), the patients with type 2 hyperlipidaemia were recruited to a group administered a complex preparation containing 170 mg of calcium lactate and 60 mg of vitamin C (Calcium C, Polfa-Lodz SA, Poland) at a dose of one tablet three times a day.

After four weeks of active therapy, the concentration of total cholesterol (TC), low-density lipoprotein cholesterol (LDL-C) and triglycerides (TG) decreased by 4, 6 and 8%, respectively. Statistical significance was obtained for only TC (*p* = 0.03) when comparing the group of patients with hypercholesterolaemia before and after the therapy with the calcium preparation. A statistically insignificant increase of high-density lipoprotein cholesterol (HDL-C) of 1% was observed. Within the four-week period of calcium supplementation at a dose of 510 mg/24 h, the total concentration of calcium decreased by 3%, whereas the concentration of ionised calcium increased by 7%. None of the obtained values was of statistical significance.

In patients with type 2 hyperlipidaemia and previous viral hepatitis, a four-week supplementation of calcium in a calcium lactate preparation beneficially modified the lipid profile. It statistically significantly decreased the total cholesterol concentration by 4% (*p* = 0.03), did not cause any significant changes in serum calcium concentration, was well tolerated and did not induce any side effects.

## Summary

Among patients with hyperlipidaemia who qualified for treatment with statins, there were some who had viral hepatitis in their history. Statins are metabolised by the liver, mainly by isoenzyme CYP3A4.^1^ Active liver disease and a three-fold increase in the activity of aminotransferases above the upper limit of the norm during the course of the therapy was a contraindication for statin therapy. However, slight elevation of the activity of aminotransferases did not disqualify the patients with a history of viral hepatitis from statin therapy, since available data showed a lack of correlation between hepatotoxicity and earlier viral infection of the liver. Gipson *et al.* administered statins to patients with chronic hepatitis C and they did not find any increase in aminotransferases.^2^ However, despite the lack of absolute contraindications for statin therapy in patients with a history of liver disease, doctors are often anxious about the safety of the therapy and eagerly use alternative methods of treatment.^3^

Decrease in total cholesterol concentrations in rats, rabbits and goats after calcium supplementation in the form of oral administration of different calcium compounds was reported in a few published experimental studies.^4^-^9^ Also in a few clinical studies, beneficial modification of lipid profiles was observed after long-term supplementation with calcium preparations.^10^-^15^

In the Polish population, calcium supply is generally lower than that required by nutritional standards and hence its supplementation is recommended.^16^ Calcium is not metabolised in the liver and therapy with calcium preparations is safe and devoid of any side effects.

The aim of the study was to estimate the effect of calcium supplementation on cholesterol concentration in patients with hyperlipidaemia and previous viral hepatitis B.

## Material and methods

The study comprised 43 patients (22 women, 21 men), aged 28 to 82 years (53.5 ± 9.25). In this group, 21 patients (nine women, aged 52.9 ± 14.92 years; 12 men, aged 48 ± 9.96 years) had a history of viral hepatitis B and at the time of the study they manifested type 2 hyperlipidaemia according to Fredrickson.^17^ Hyperlipidaemia was diagnosed on the basis of laboratory tests the patients had on admission to the hospital and then again at our department for confirmation.

Initial concentrations of TC > 200 mg/dl (5.2 mmol/l), LDLC > 145 mg/dl (3.75 mmol/l) and TG < 400 mg/dl (4.54 mmol/l) were the criteria for inclusion into the patient group. The control group included 22 healthy subjects (nine men; 13 women, aged 56.9 ± 6.3 years) with normal lipid values. Patients with other types of hyperlipidaemia, with obesity (BMI > 30 kg/m^2^), and renal and liver failure were excluded from the study.

There were no smokers, people abusing alcohol, or taking anticoagulants, cardiac glucosides or hypolipaemic drugs among the tested patients. Seven patients (four women and three men) had a diagnosed mild hypertension (stage 1 according to WHO) and they were taking one drug on a regular basis (indapamide, perindopril, lisinopril, enalapril, potassium losartan or nitrendipine). During subsequent follow up, the blood pressure values were in the normal range.

The study schedule included a four-week diet limiting the fat content (wash-out period) prior to the examination. In patients in whom the values of TC, LDL-C and TG exceeded the given reference ranges, the treatment was introduced with a complex preparation containing 170 mg calcium lactate and 60 mg vitamin C (Calcium C, Polfa-Lodz SA, Poland) in the form of effervescent tablets. The recommended dose was one tablet three times daily with meals. Active supplementation lasted four weeks.

The examined subjects were allocated into two groups: group 1: 21 patients with a history of viral hepatitis B and type 2 hyperlipidaemia (nine women and 12 men), aged 50.4 ± 14.9 years); control group 2: 22 healthy subjects (nine men and 13 women) with normal lipid concentrations, aged 56.90 ± 6.3 years).

Fasting blood was collected from a cubital vein (at least 14 h after the last meal). The determination was performed prior to the examination and after four weeks of therapy. In the control group, the tests were carried out once during periodic medical examinations. A lipid profile was done and the concentration of total and ionised calcium was determined in each examined subject.

Biochemical investigations were done on a Cobas Integra 800, Hitachi (Switzerland) using Roche kits. TC, LDL-C, HDLC and TG were determined by enzymatic methods with Elecsys 2010 (Japan) and Roche kits. LDL-C concentrations were calculated according to Friedwald’s formula.

Approval of the Medical University of Lodz Ethics Committee for Scientific Research was received for this study (No RNN30/05/KB). Written, informed consent from each patient was obtained before the study.

For statistical analysis, Smirnow’s test was used to assess the distribution of variables. When the distribution of the investigated ranks was in accordance with a normal distribution, the Student’s *t*-test was applied. When the distribution of ranks was not in accordance with a normal distribution, the Wilkoxon’s test for matched pairs and Mann-Whitney *U*-test for unrelated pairs were used.

## Results

**Fig. 1.**
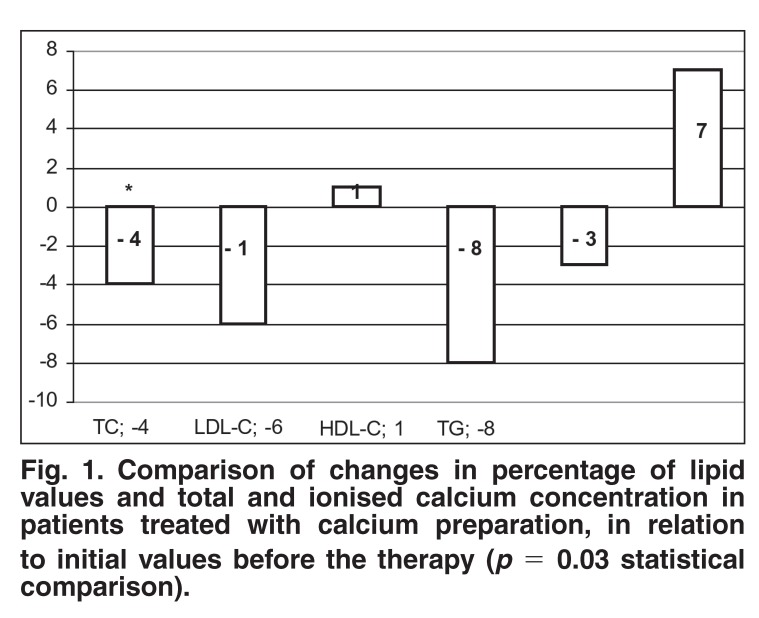
Comparison of changes in percentage of lipid values and total and ionised calcium concentration in patients treated with calcium preparation, in relation to initial values before the therapy (*p* = 0.03 statistical comparison).

After four weeks of active therapy with the calcium lactate preparation, the concentration of TC, LDL-C and TG decreased by 4, 6 and 8%, respectively. Statistical significance was obtained for only TC (*p* = 0.03) when comparing the group of patients with hypercholesterolaemia before and after the therapy. The other lipid fractions (LDL-C and TG) did not demonstrate any statistical significance. Statistically insignificant increases of HDL-C of 1% (*p* > 0.05) were observed. Within the four-week calcium-supplementation period (total dose of 510 mg/24 h), total calcium concentrations decreased by 3%, whereas the concentration of ionised calcium increased by 7%. None of the obtained values was of statistical significance. The detailed results are shown in Fig. 1 and Tables 1, 2, 3.

**Table 1.** Characteristics Of Patients

		*Investigated group (n = 21)*	
*Parameters*	*Control group (n = 22)*	*Before therapy with calcium preparation*	*After therapy with calcium preparation*
Age	56.9 ± 6.30	50.09 ± 12.23	50.09 ± 12.23
BMI	25.3 ± 3.6	26.6 ± 3.1	25.2 ± 3.7
Number of current smokers	5	2	2
Hypertension			
women	0	4	4
men	0	3	3
Arterial pressure	127.2 ± 10.7	126.7 ± 11.8	125.8 ± 9.8
Transaminases activity (U/l)			
ALT	23.31 ± 10.2	25.85 ± 18.65	25.02 ± 17.07
AST	22.73 ± 6.75	23.35 ± 8.08	23.26 ± 7.62
Urea (mg/dl)	32.76 ± 6.74	31.52 ± 5.4	33.35 ± 6.25
Creatinine (mg/dl)	0.81 ± 0.11	0.9 ± 0.19	0.83 ± 0.18

**Table 2.** Mean Values Of Serum Lipid Concentrations In Patients After Therapy With Calcium Preparation And In Healthy Subjects (± SD; MIN−MAX)

	*Control group (n = 22) ± SD (min−max)*	*Before therapy (n = 21) ± SD (min−max)*	*After therapy (n = 21) ± SD (min−max)*
TC (mg/dl)	189.1 ± 15.16 (146.0−204.0)	249.8 ± 37.94 (203−338)	240.28 ± 35.44* (181−308)*
LDL-C (mg/dl)	119.3 ± 12.11 (90.0−134.0)	165.95 ± 35.98 (106−261)	157.57 ± 31.79 (120−234)
HDL-C (mg/dl)	50.0 ± 10.54 (32.1−69.8)	53.78 ± 16.91 (34−90.3)	53.86 ± 17.62 (32.5−94.7)
TG (mg/d)	100.1 ± 25.4 (52.0−147.0)	159.09 ± 111.22 (52−396)	146.8 ± 75.31 (40−381)

*p = 0.03 statistical comparison in relation to initial values before the therapy with calcium preparation

**Table 3.** Mean Values Of Total And Ionised Calcium Concentration Before And After Therapy With Calcium Preparation (± SD; MiN−MAX)

	*Control group (n = 22) ± SD (min−max)*	*Before therapy (n = 21) ± SD (min−max)*	*After therapy (n = 21) ± SD (min−max)*
Total Ca (mg/dl)	9.3 ± 0.22 (8.84−9.96)	9.16 ± 1.07 (5−10.25)	8.97 ± 1.41 (4.87−10.63)
Ionised Ca (mg/dl)	4.34 ± 0.22 (4.06−4.91)	3.98 ± 0.92 (0.98−4.6)	4.27 ± 0.23 (3.91−4.78)

The activity of aminotransferases was determined in all the examined patients before the introduction of the therapy. In four patients with a history of viral hepatitis B, the values of ALAT and AST were elevated insignificantly. They did not exceed twice the upper normal limit in any case. No statistically significant difference was observed in the activity of aminotransferases in patients before and after the therapy with the calcium preparation.

## Discussion

In the available literature, a few clinical studies have been described in which a decrease in cholesterol level or its fraction was observed during the course of calcium supplementation. In one of the studies, during an eight-week calcium supplementation, the decrease in cholesterol concentration from 8.30 to 7.84 mmol/l (321−303 mg/dl) was observed in 16 patients.^11^

In another study of 13 volunteers, a decrease in cholesterol of 0.06 mmol/l (0.2 mg/dl) was detected in the control group, whereas in patients who were administered calcium, it decreased by 0.61 mmol/l (23.6 mg/dl) in healthy subjects and by 34.5% in patients with hypercholesterolaemia.^12^

In a double-blind trial of 20 patients with hyperlipidaemia, mean levels of cholesterol decreased from 9.03 to 7.19 mmol/l (349−278 mg/dl) within six months and to 6.78 mmol/l (226 mg/dl) within 12 months. The patients received calcium carbonate in a dose of 2.0 g/24 h. In the control group, mean cholesterol concentration decreased by only 0.1 mmol/l (4 mg/dl).^13^ In each trial, a significant decrease of TC was observed.

The authors found a positive effect of calcium supplementation on the treatment of patients with hypercholesterolaemia. The presented studies are from the years 1965, 1971 and 1972, which is the period before statins and fibrates were discovered. This makes the importance of this discovery significant. A limitation was the small groups of patients included in these studies.

The results of a study published in 2002 concerned 223 postmenopausal women. The investigated group of 111 women received calcium citrate in a dose of 1 g/24 h. After 12 months of active calcium supplementation, a statistically significant increase in HDL cholesterol of 7% (*p* = 0.01) and a statistically insignificant decrease in LDL fraction and triglycerides were observed. The authors of the study concluded that postmenopausal women should be supplemented with calcium as it has a positive effect on cholesterol and its fractions.^14^

Our own observations carried out on a group of 43 patients with type 3 hyperlipidaemia confirmed that a four-week supplementation with calcium lactate preparation and vitamin C had a positive effect on the lipid profiles of those examined. It was the first study using a complex calcium lactate preparation. A significant decrease in total cholesterol of 4% was demonstrated in these investigations. Each patient received one tablet of the calcium preparation three times daily. During the second check-up, the number of consumed tablets was controlled. In comparison with other studies, the total amount of administered calcium was small, as it was a dose of only 510 mg of calcium lactate. The effectiveness of this preparation probably resulted from good solubility of calcium lactate in aqueous medium, and the increased bioavailability of vitamin C found in the preparation.^18^

The mechanism of the effect of calcium on the concentration of cholesterol and its fractions in blood serum is little known. In the alimentary tract, calcium probably binds with bile acids and the cholesterol contained in food. This causes precipitation of insoluble salts, which are not absorbed from the alimentary tract into the blood stream, and are excreted. Increased cholesterol catabolism may cause induction of the LDL receptor. During the four-week therapy with calcium lactate, no side effects were reported, no disorders were found in renal efficiency (creatinine, urea) or liver function (ALAT, AspAT).^19^,^20^

Due to the small number of patients, the short period of treatment and the relatively low dose of calcium lactate, the presented outcomes should be considered as a preliminary study. Further studies (being continued by the authors) are necessary to confirm the role of calcium lactate preparation in this selected group of patients.

## Conclusion

In patients with type 2 hyperlipidaemia and a history of viral hepatitis, a four-week calcium lactate supplementation: (1) beneficially modified lipid profiles − decreased total cholesterol by 4% (*p* = 0.03); (2) did not cause significant changes in serum calcium concentrations; and (3) was well tolerated and did not induce any side effects. A longer observation period is required to estimate the effects of long-term calcium lactate supplementation on lipid profiles of patients with hyperlipidaemia.
